# RedOx Status, Proteasome and APEH: Insights into Anticancer Mechanisms of t10,c12-Conjugated Linoleic Acid Isomer on A375 Melanoma Cells

**DOI:** 10.1371/journal.pone.0080900

**Published:** 2013-11-19

**Authors:** Paolo Bergamo, Ennio Cocca, Rosanna Palumbo, Marta Gogliettino, Mose Rossi, Gianna Palmieri

**Affiliations:** 1 Institute of Food Sciences, National Research Council (CNR-ISA), Avellino, Italy; 2 Institute of Protein Biochemistry, National Research Council (CNR-IBP), Napoli, Italy; 3 Institute of Biostructure and Bioimaging, National Research Council (CNR-IBB), Napoli, Italy; Duke University Medical Center, United States of America

## Abstract

This study describes the investigation of the efficiency of conjugated linoleic acid (CLA) isomers in reducing cancer cells viability exploring the role of the oxidative stress and acylpeptide hydrolase (APEH)/proteasome mediated pathways on pro-apoptotic activity of the isomer trans10,cis12 (t10,c12)-CLA. The basal activity/expression levels of APEH and proteasome (β-5 subunit) were preliminarily measured in eight cancer cell lines and the functional relationship between these enzymes was clearly demonstrated through their strong positive correlation. t10,c12-CLA efficiently inhibited the activity of APEH and proteasome isoforms in *cell-free* assays and the negative correlation between cell viability and caspase 3 activity confirmed the pro-apoptotic role of this isomer. Finally, modulatory effects of t10,c12-CLA on cellular redox status (intracellular glutathione, mRNA levels of antioxidant/detoxifying enzymes activated through NF-E2-related factor 2, Nrf2, pathway) and on APEH/β-5 activity/expression levels, were investigated in A375 melanoma cells. Dose- and time-dependent variations of the considered parameters were established and the resulting pro-apoptotic effects were shown to be associated with an alteration of the redox status and a down-regulation of APEH/proteasome pathway. Therefore, our results support the idea that these events are involved in ROS-dependent apoptosis of t10,c12-CLA-treated A375 cells. The combined inhibition, triggered by t10,c12-CLA, *via* the modulation of APEH/proteasome and Nrf2 pathway for treating melanoma, is suggested as a subject for further *in vivo* studies.

## Introduction

Oxidative stress is a dynamic status characterized by an imbalance between the production of reactive oxygen species (ROS) and the activity and availability of antioxidants. Organisms have developed a hierarchy of defence strategies to deal with oxidative stress in which antioxidants (molecules and enzymes) provide the first defensive mechanism, and proteolytic systems act as secondary defences [1]. Among intracellular antioxidants, reduced glutathione (GSH) plays a central role in the maintenance of the thiol-disulfide redox state in mammalian cells and its deregulation is responsible for apoptosis evasion [2], colonizing ability [3] and multidrug resistance of cancer cells [4]. Interestingly, alterations in redox status are known to lead the induction of apoptosis in cancer cells and its decrease represents a molecular mechanism whereby anti-cancer agents reduces malignant cell survival [5].

The proteasome is a multi-catalytic protease responsible for intracellular protein degradation and dysregulation of its activity has been implicated in the pathogenesis of many diseases, including cancer. Proteasome inhibition has recently emerged as an attractive target for anticancer therapy [6]; the rationale for such targeting arose from the concept that in cancer cells, likely because of their higher metabolic rate, proteasome functionality is more crucial than in untransformed cells. Of note, during oxidative stress, the higher activity of 20S proteasome core enzymes aimed at counteracting the accumulation of oxidatively damaged proteins, greatly contributes to secondary anti-oxidative defences [1]. However, a delicate balance between cellular redox status and proteasome activity is clearly indicated by ROS production during the initiation of apoptotic signalling triggered by bortezomib (BTZ, Velcade), a widely used proteasome inhibitor [7-10] and the impairment of proteasome activity by oxidative stress [11,12].

Due to substrate specificity, 20S enzymes only cleave a limited percentage of peptide bonds in proteins [13] and complete conversion to amino acids is carried out by cytosolic exo- and endo-peptidases which play an important role in cleaving proteasomal produced peptides [14]. Among these proteases, acylpeptide hydrolase (APEH), also named Acylaminoacyl Peptidase or Oxidised Protein Hydrolase, catalyses the removal of N-acylated amino acids from acetylated peptides and was hypothesized to participate in the coordinated degradation of oxidatively modified proteins [15,16].

Conjugated linoleic acid (CLA) is a collective term used to describe the positional and geometric isomers of this fatty acid. Among the eight possible isomers, cis9,trans11 (c9,t11-CLA) and trans10,cis12 (t10,c12-CLA) have attracted considerable attention for their putative health beneﬁts [17]. The commercially available CLA mixture, containing approximately equal amounts of these isomers, exhibited antitumor activity against a broad range of cancer cell types [18] and hindered the growth of numerous types of tumors [19,20]. Of note, similarly to proteasome inhibitors, in several studies the pro-oxidant activity of CLA was associated to its pro-apoptotic effects on cancer cells [21-23] and the modulatory ability of CLA on APEH and proteasomal chymotrypsin-like (CT-like) activities was demonstrated [24,25]. In addition, CLA ability to influence redox status through the activation of NF-E2-related factor 2 (Nrf2) *in vivo* was recently demonstrated [24,26]. The dissociation of Nrf2 from the Kelch-like (Keap1) triggered by electrophile or oxidative stress induces its nuclear translocation and the down-stream activation of genes coding for highly specialized antioxidant and detoxifying proteins. In addition, although Nrf2 has been indicated as a potential target in anticancer therapy [27], nevertheless, to our knowledge, its involvement in the anticancer activity of CLA has not been yet investigated. 

Herein, the cellular redox status along with the reduced cancer cell viability induced by CLA isomers, were investigated in relation to the APEH/proteasome system. To this aim, we examined the basal expression/activity level of proteasome and APEH and the anti-proliferative activities elicited by three different CLA isomers (c9,t11-, t9,t11- or t10,c12-CLA) in eight randomly selected cancer cell lines. Interestingly, we identified APEH/proteasome and Nrf2 pathways as the key factors involved in the pro-apoptotic effects of t10,c12-CLA on the A375 melanoma cell lines, revealed to be the best suited experimental model.

## Materials and Methods

### Materials

Pure fatty acids (octanoic acid, c9,t11-, t9,t11- and t10,c12-CLA isomers), caspase 3 fluorometric assay kit were from Sigma-Aldrich. DMEM, L-glutamine, penicillin-streptomycin and fetal bovine serum (FBS) were from Gibco-BRL. Porcine liver APEH was obtained from Takara. 20S, 20Si, 26S human proteasome were purchased from Boston Biochem. Bortezomib (BTZ) was obtained by Santa Cruz Biothecnology. The following antibodies were used: anti-APEH antibody (sc-102311; Santa Cruz Biotechnology); pan Ab-5 anti-actin antibody (clone ACTN05, Thermo Scientific); anti-Bcl-2 (340576-BD Pharmingen^TM^); anti-proteasome 20S β-5 subunit (BML-PW8895-0025; Enzo Life Science). All chemicals were obtained from Sigma-Aldrich or Calbiochem.

### Enzyme assays

APEH activity was measured spectrophotometrically using the chromogenic substrate acetyl-Ala-pNA (Bachem) as described before [25]. The reaction mixture containing pure APEH or an appropriate amount of cell extract was incubated at 37 °C in 50 mM Tris-HCl, pH 7.5 (Tris Buffer).

The fluorescent substrate N-succinyl-Leu-Leu-Val-Tyr-7-amido-4-methylcoumarin (N-Suc-LLVT-AMC) was used for measurement of the CT-like activity of the proteasome, both in *cell free* assays and in cancer cell extracts, at a final concentration of 0.080 mM at 37 °C in Tris buffer pH 7.5, following the procedure described in Palmieri et al. [25]. 

### Enzyme inhibitory assays

Protease inhibition activities of CLA isomers and fatty acids were carried out using a fixed amount of commercially available APEH or 20S, 20Si, 26S proteasomes (3-5 nM or 0.12 mg/mL, respectively), and increasing concentrations of fatty acid. Mixtures were pre-incubated for 30min at 37 °C in 50 mM Tris-HCl buffer pH 7.5, before addition of the specific substrate, and the enzymatic activities were followed as described above. 

Inhibitory *cell free* assays were also performed on APEH and proteasome partially purified from A375 cells at 37 °C in Tris buffer pH 7.5 according to a published protocol [25] and the IC_50_ (concentration required for obtaining 50% of the maximum effect measured) values were determined (data not shown). These values, using the considered CLA isomers, were comparable to those obtained with the commercially available porcine APEH (sharing more than 90% of sequence identity with human APEH as calculated by the ClustalW algorithm http://www.genome.jp/tools/clustalw/) and human proteasomes, therefore these enzymes were hereafter used in this study.

### Cells, culture conditions and treatments

Human hepatoma cells (HepG2) were seeded (2x10^4^cells/cm^2^) and maintained for 24h in MEM (Gibco Invitrogen; Milano) medium supplemented with 2 mM L-glutamine, 1% nonessential amino acids and 10% FBS. Colon carcinoma (Caco-2), cervical carcinoma (Hela), glioblastoma (U87), melanoma (A375, A375M) and mammary adenocarcinoma (MCF7, MDA-MB) were seeded (1x10^4^cells/cm^2^) and grown in DMEM supplemented with 10% FBS, 2 mM L-glutamine. HepG2, Hela, U87, Caco-2, MCF7, MDA-MB, A375 and A375 metastatic melanoma (A375M) cell lines were obtained by ATCC (LCG standards, Milano, Italy). Normal human dermal fibroblast (NDHF) within 8^th^ passage were seeded at a density of 2x10^4^ cells/cm^2^ and cultured in fibroblast growth medium (FGM-2; Lonza, Milan, Italy) containing 2% FBS, 50 μg/mL gentamycin and amphotericin B, 10μg/mL fibroblast growth factor and insulin. BHK21 cells (kindly donated by Dr. David Y Thomas, McGill University Montreal Canada) were cultured in DMEM/F12, 5% FBS, 1 mM L-glutamine, 200 µg/mL methotrexate, and 100units/mL penicillin-streptomycin. Cells were incubated in a humidified atmosphere at 37 °C in 5% CO_2_. 

Stock solutions of fatty acids or bortezomib (BTZ) were prepared by dissolving in dimethyl sulphoxide (DMSO) to the final concentration of 1 M or 8.6 mM, respectively, and further dilutions were carried out in DMEM. Cells were treated with fatty acids or BTZ and control culture were exposed to the same amount of DMSO. 

### MTT-based cytotoxicity assay

The colorimetric 3-(4,5-dimethylthiazol-2)-2,5-diphenyltetrazolium bromide (MTT) (Sigma Aldrich, Milan) assay was used to quantify cell viability. Briefly, cells were incubated in 96 well microplates in the appropriate complete medium with standardized densities for 24h as pre-incubation process. The medium was removed and replaced by medium containing different doses of the different CLA-isomers for 24h. Following treatment, the medium was removed and the cells were incubated with DMEM w/o red phenol with 0.5 mg/ml MTT for additional 2 to 4h at 37 °C. After removal of the medium and MTT, cells in each plate were incubated with 0.1 M HCl/isopropanol to dissolve the MTT-formazan crystals. Absorbance at 590 nm was recorded with a plate reader (Bio Rad mod 680). The relative number of viable cells was expressed as a percentage of the control.

### Cytosolic cell extracts and Western blotting analysis

Following the treatments, cells were washed three times with ice cold phosphate-buffer saline (PBS) and cytosolic extracts were prepared accordingly to a published procedure [26]. Protein concentrations were determined in supernatants by BCA protein assay reagent kit (Pierce). Western blotting analyses were performed as previously described [25].

### Intracellular redox status and cell viability assessment

Intracellular concentration of reduced and oxidized glutathione (GSH and GSSG, respectively) were quantified using the 5,5'-dithiobis(2-nitrobenzoic acid)-GSSG reductase recycling assay [26]. GSSG content was preliminarily evaluated in cytosolic extracts of treated or untreated cells upon the addition of 10 mM 1-methyl-2-vinylpyridinium trifluoromethanesulfonate (a specific GSH scavenger). Notably, owing to the minor contribution given by GSSG (less than 5%) to the total intracellular thiol concentration, the latter was finally expressed as nmol GSH/mg protein. 

Pro-apoptotic and cytotoxic ability of CLA isomers were assayed by measuring caspase 3 and the activity of lactate dehydrogenase (LDH) in the spent media, respectively [25]. The caspase 3 activity, measured at 37 °C and pH 7.5, was expressed as fold increase compared to the control culture. The LDH release, measured at 37 °C and pH 8.2, was expressed as percentages of total LDH released from cultures treated with 1% (w/v) Triton X-100 and calculated as: [(experimental value-blank value)/(total lysis-blank value)-100].

### ROS detection

DCF-DA (2’,7’-dichlorofluorescein diacetate) was used to determine the amount of ROS production. DCF-DA working solution was added to the medium to reach 10 μM and then incubated at 37 °C for 15min in the dark. Cells were harvested by trypsinization, washed with PBS and kept on ice for detection by FACScan (Becton Dickinson, USA) equipped with a 488 nm argon laser using a band pass filter of 530 nm.

### RNA isolation and quantitative real-time PCR analysis

mRNA expression levels of APEH and β-5 proteasome subunit were determined in treated or untreated cells to investigate on the functional relationship existing between APEH and proteasome activities and on their involvement in the anticancer activity of CLA. In addition, the mRNA expression of NADH quinone oxidoreductase (NQO1) and of gamma Glutamylcysteine Ligase (γGCL), which is the limiting enzyme in the GSH synthesis, were also measured to further demonstrate the CLA ability to down-regulate intracellular redox status via the Nrf2 pathway. 

Total RNA was isolated from the human cell lines (~10^6^ cells aliquots) according to the SV Total RNA Isolation System (Promega) protocol, with an on column DNase I step. Total RNA concentrations were determined using a Qubit® Fluorometer (Invitrogen). RNAs were then reverse transcribed using the Transcriptor First Strand cDNA Synthesis Kit (Roche). 100 ng of reverse transcribed complementary DNA, and its dilution series to calculate the efficacy of primers, were amplified by quantitative real-time PCR (qRT-PCR) on an iCycleriQ™ (Bio-Rad) using 300 nM gene-specific primers, Maxima® SYBR Green/Fluorescein qPCR Master Mix (2X) (Fermentas) and the following PCR conditions: 1 cycle at 95 °C for 10min, and 40 cycles of 95 °C for 15s , 60 °C for 30s, and 72 °C for 30s. 

The expression level of β-actin gene was used as an internal control for normalization (ref gene). Raw cycle threshold values (Ct values) obtained for the target genes were compared to the Ct value obtained for the ref gene. The final graphical data were derived from the R=(E_target_)^ΔCt_target (control - sample^)/(E_ref_)^ΔCt_ref (control - sample)^ formula [28], where “control” cells were fibroblasts or A375 line, and “sample” cells were the tumor lines. In time-course analysis the expression levels were normalized to those of untreated cells at Time=0. 

Universal Probe Library Assay Design Center (https://www.roche-appliedscience.com/sis/rtpcr/upl/index.jsp?id=UP030000) was used for designing primers. 

The primers utilized were: 

APEH, 5’-CCCCATTCATCCTTTGTCAC-3’ and 5’-AAAGCCCATCTTGCAAAGC-3’; 

β-5, 5’-CATGGGCACCATGATCTGT-3’ and 5’-GAAATCCGGTTCCCTTCACT-3’; 

γGCL, 5’-GACAAAACACAGTTGGAACAGC-3’and 5’- CAGTCAAATCTGGTGGCATC-3’; 

NQO1, 5’-CAGCTCACCGAGAGCCTAGT-3’ and 5’-GAGTGAGCCAGTACGATCAGTG-3’;

β-actin, 5’-CCAACCGCGAGAAGATGA-3’ and 5’-CCAGAGGCGTACAGGGATAG-3’.

### Statistical analysis

All data were obtained from triplicate analyses of three different preparations, and presented as means ±S.D. Statistical analysis and IC_50_ values were calculated with SigmaPlot 10.0 software through a non-linear curve-fitting method and using a simple binding isotherm equation. Groups were compared by Student’s *t* test, and *P*<0.05 was considered as significant.

## Results

### t10,c12-, t9,t11- and c9,t11-CLA isomers differentially inhibit APEH and proteasome

**Figure 1:**
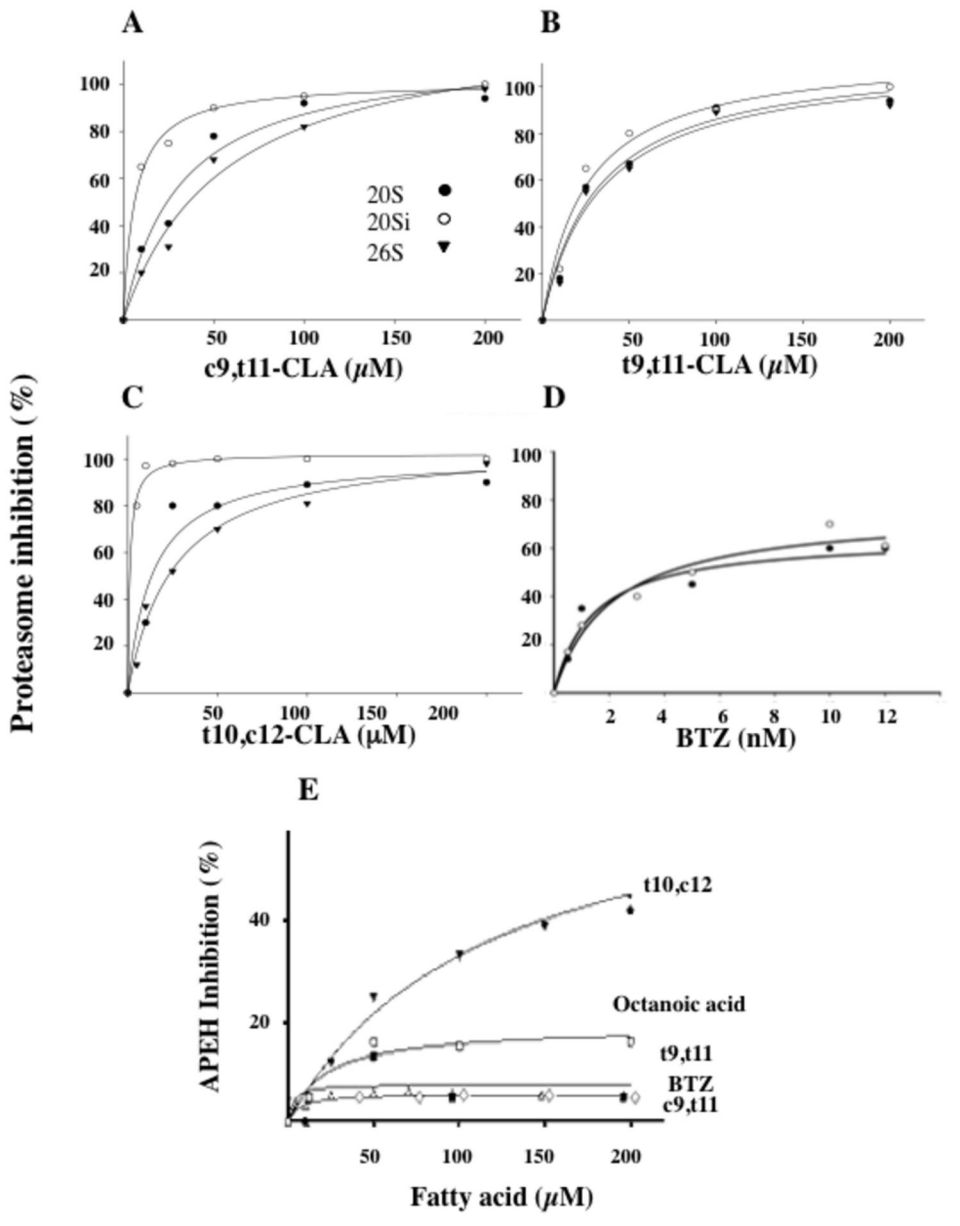
Fatty acids and CLA isomers exhibit dissimilar inhibitory ability towards chymotrypsin-like (CT-like) proteasome and APEH activities. The inhibitory effect of different CLA isomers, namely c9,t11- (**A**), t9,t11- (**B**), t10,c12-CLA (**C**), bortezomib, BTZ (**D**), was evaluated on commercially available pure 20S (black circles), 20Si (white circles) and 26S (black triangles) proteasomes. The synthetic fluorescent substrate N-Suc-LLVT-AMC (0.080 mM) was used for the measurement of the CT-like activity of the proteasomes. The hyperbolic curves indicate the best fits for the data obtained, with IC50 values calculated from the graphs by SigmaPlot 10.0 software. Mixtures treated with DMSO alone were used as blank. The dose-dependent inhibitory effect of c9,t11-, t9,t11-, t10,c12-CLA isomers, octanoic acid or bortezomib on APEH activity was shown (**E**). Results are presented as the mean ± standard deviation (SD) of triplicate analyses from three independent experiments. SD values lower than 5% were not shown.

A preliminary investigation of the potential inhibitory effect of CLA isomers on chymotrypsin-like (CT-like) activity of proteasome isoforms (20S, 20Si and 26S), was carried out. Inhibition analyses were performed by pre-incubating the purified enzyme with increasing amounts of each compound and their half-maximal inhibitory concentrations (IC_50_) were determined. The curves followed a hyperbolic pattern reaching 100% inhibition with all the proteasomes (Figure 1A-C) and CLA isomers tested, althought t10,c12- and c9,t11-CLA were the best effectors (IC_50_=14.8±2.0 μM and 31.2±8.8 μM on 20S isoform and 1.1±0.2 μM and 6.4±1.0 μM on 20Si isoform for t10,c12- and c9,t11*-*CLA, respectively). Similar experiments were carried out by using the proteasome inhibitor bortezomib (BTZ) (Figure **1D**) because of its recognized anti-proliferative activity on cancer cells. As expected, BTZ appeared to target both 20S (IC_50_=1.4±0.3 nM) and 20Si (IC_50_=2.1±0.5 nM) isoforms indiscriminately, reaching about 60% of inhibition in both cases, while octanoic acid (data not shown), used as a negative control, gave only negligible effects. 

Next, before to investigate the possible mechanisms underlying the CLA-reduced viability of cancer cells, the potential contribution of APEH was explored. When the ability of these compounds to modulate APEH in *cell-free* assays was evaluated (Figure **1E**), only t10,c12-CLA was revealed to affect the enzyme activity in a dose-dependent manner, reaching a maximum inhibition of about 41% (IC_50_=110.1±11.7 μM) as calculated by SigmaPlot 10.0 software. 

Therefore, a stereoselective binding in the interaction with APEH and proteasome isoforms of the CLA isomers can be proposed together with a specific ability of t10,c12-CLA to inhibit all these enzymes . 

### APEH and proteasome expression at both mRNA and protein level correlates with their enzyme activity in cancer cell lines

**Figure 2:**
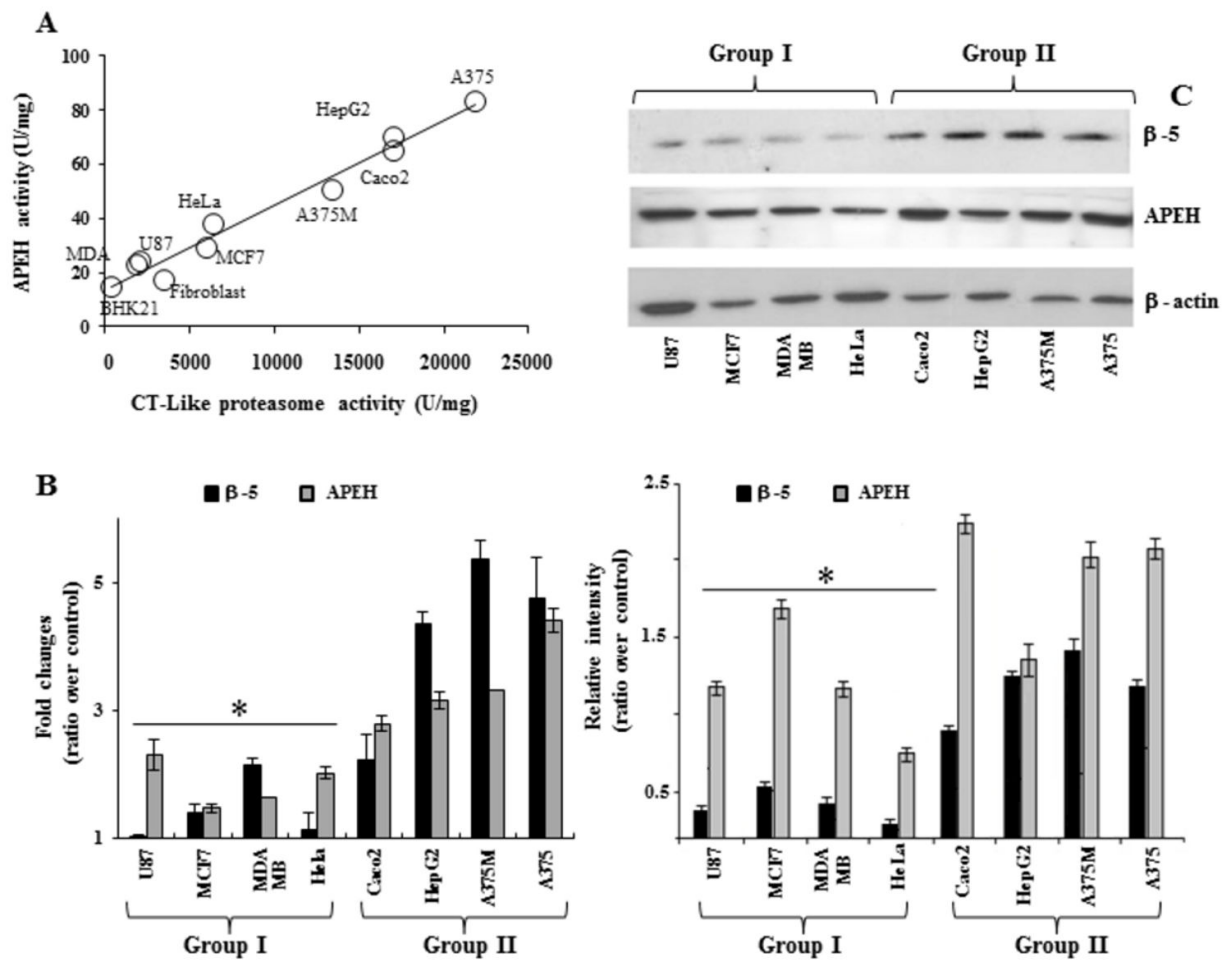
Human cancer cell lines may be grouped according to the basal enzyme activities and expression levels of APEH and proteasome. Cells from eight human cancer lines (U87, MCF7, MDA-MB, HeLa, Caco_2_, HepG2, A375M, A375) and non-cancerous cells (BHk21, fibroblasts) were harvested at the pre-confluent stage and used for cytoplasmic or mRNA extract preparation. Basal APEH and proteasomal CT-like activities were measured in cytoplasmic extracts (A). The mRNA levels of APEH and β-5 subunit were evaluated by qRT-PCR and expressed as fold change in comparison to expressed levels in human fibroblast (B). Intracellular levels of β-5 and APEH were detected by immunoblotting (C upper panel). Typical Western blot was shown and data from three different analyses were normalized to the density of control protein (β-actin) and expressed as ratio over control (C lower panel). Results are presented as the mean values ±SD of triplicate analyses from at least three different experiments. *Significantly different (P < 0.01) from respective controls.

In evaluating the involvement of APEH and proteasome in the anti-cancer effects of CLA isomers, we decided to examine the basal expression/activity levels of these enzymes in eight cancer cell lines (at their pre-confluent stage) to select the best cellular candidate for further investigations. As shown in Figure 2A, when basal specific APEH activity was plotted against the corresponding proteasomal chymotrypsin-like (CT-like) activity, a significant positive correlation was found (r^2^=0.988, P=<0.01), supporting the idea of a functional relationship between these two enzymes which could act in cooperation for degradation of damaged proteins [15,16]. Moreover, on the basis of gene expression analysis and intracellular protein levels (Figure 2B and 2C), the different cancer cells could be divided into two groups displaying low (U87, HeLa, MDA-MB and MCF7: Group I) or high (A375, A375M, HepG2 and Caco_2_: Group II) protein, activity and transcript levels of APEH and proteasome (β-5 subunit). Data on the immunoproteasome subunit (β-5i subunit) were not reported due to the low detectable levels in all the cancer cells investigated. These findings suggested that cells exhibiting high basal activity and expression levels of APEH and proteasome could be highly dependent on these enzyme functions and therefore more sensitive to their down-regulation.

### Cancer cell proliferation is significantly inhibited and associates with caspase 3 activation in cells exposed to t10,c12-CLA

**Figure 3:**
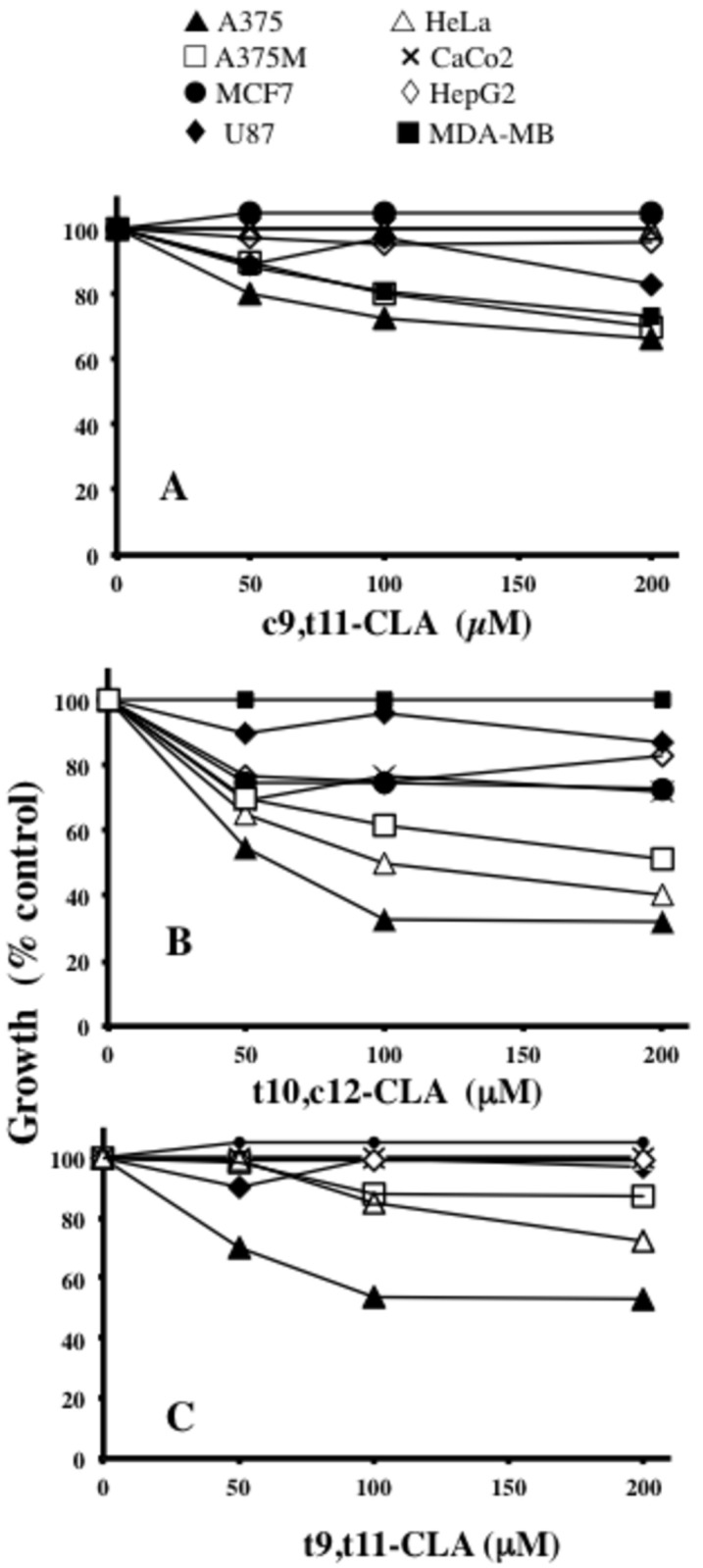
Human cancer cells exhibit differential sensitivity to the anti-proliferative activity of CLA isomers. The effects of c9,t11 (A), t10,c12- (B) or t9,t11-CLA isomers (C) on cell viability were assessed in eight cancer cell lines exposed for 24h to increasing concentrations of the CLA isomers. Data are expressed as means ±SD values of triplicate data from three independent experiments. SD values lower than 5% were not shown.

The susceptibility of cell lines belonging to Group I and Group II to the growth inhibitory effects of CLA isomers was estimated upon 24h exposure at concentrations ranging from 50 to 200 μM. Data indicated that only A375, A375M and MDA-MB cells exhibited a moderate reduction (<40%) of cell viability by c9,t11-CLA treatment (Figure 3A), while a more marked anti-proliferative effect was observed following cell exposure with t10,c12-CLA on HeLa, A375M and A375 (40, 51 and 63% respectively) (Figure 3B). A375 cell viability was also greatly influenced by t9,t11-CLA (about 50%) (Figure 3C), whereas no significant results were obtained on all cancer cells by octanoic acid (up to 200 μM) treatment, which was used as a negative control (data not shown). 

**Figure 4:**
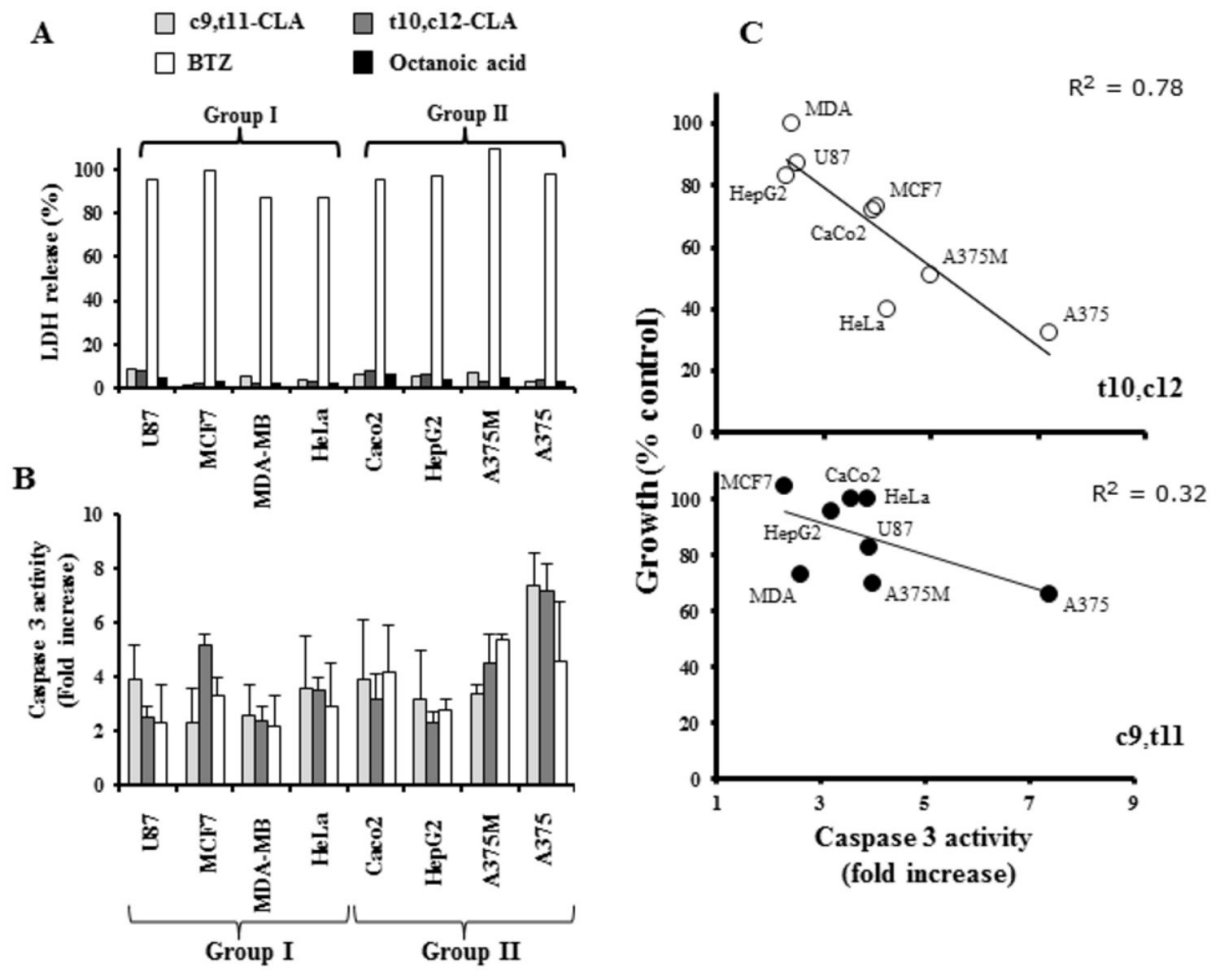
Anti-proliferative ability of t10,c12-CLA correlates with caspase 3 activation. LDH release (A) and caspase 3 activity (B) were measured to study the cytotoxic and pro-apoptotic ability of 200 µM of t10,c12- (dark grey bars) or c9,t11-CLA (light grey bars). Cell cultures exposed to octanoic acid (200 µM, black bars) or to BTZ (10 nM, white bars) were used as negative or positive controls, respectively. Average caspase 3 activity values (fold increase) in cancer cells exposed for 24h to 200 µM t10,c12- (C upper panel) or to c9,t11- CLA (C lower panel) were plotted against cell viability (%).

To assess the cytotoxicity and antiproliferative activity of the most abundant CLA isomers on the cancer cells considered, LDH activity was measured in spent media following 24h exposure to 200 μM c9,t11-, t10,c12-CLA or to 10 nM BTZ, using octanoic acid as negative control. As expected, substantial cell death resulted from BTZ supplementation while the LDH activity in cultures exposed to CLA isomers was comparable to that of control (Figure 4A). Moreover, to examine the contribution of an apoptotic event in CLA-induced decline of cancer cells viability, caspase 3 activation was measured. Interestingly, results revealed that while caspase 3 activation varied slightly between the different tumor cell lines upon exposure with c9_,_t11-CLA, a more marked variation was observed by t10,c12-CLA treatment (Figure 4B), leading to inversely correlated measures of cell viability and caspase 3 activation (r^2^=0.78; P<0.01) (Figure 4C). It’s worth to note that, although CLA reduced cell viability in the considered cell lines with no cytotoxicity (LDH release), nevertheless its pro-apoptotic activity couldn’t be accounted for the observed cell death, therefore a cytostatic effect cannot be excluded.

In addition, proteasome activity was differently down-regulated by CLA isomers (Figure S1) but it was not significantly correlated with cell viability decrease (r^2^=0.046; data not shown), suggesting that proteasome inhibition alone was not liable for the observed anti-proliferative activity of CLAs. Hence, it appears reasonable to hypothesize that an enzyme machinery, such as APEH/proteasome system, could be involved in the marked anti-proliferative and pro-apoptotic activity exerted by t10,c12-CLA through  its specific capacity to down-regulate both enzymes (Figure 1).

### t10,c12-CLA decreases glutathione level and APEH/proteasome activity in A375 cells triggering apoptosis in a dose-dependent fashion

On the basis of the marked cell viability reduction (Figure 3B) induced by t10,c12-CLA on A375 melanoma cell line, we decided to use this model system for investigations on the different cellular factors (redox status, caspase 3, APEH and proteasome) involved in the apoptotic pathway.

**Figure 5:**
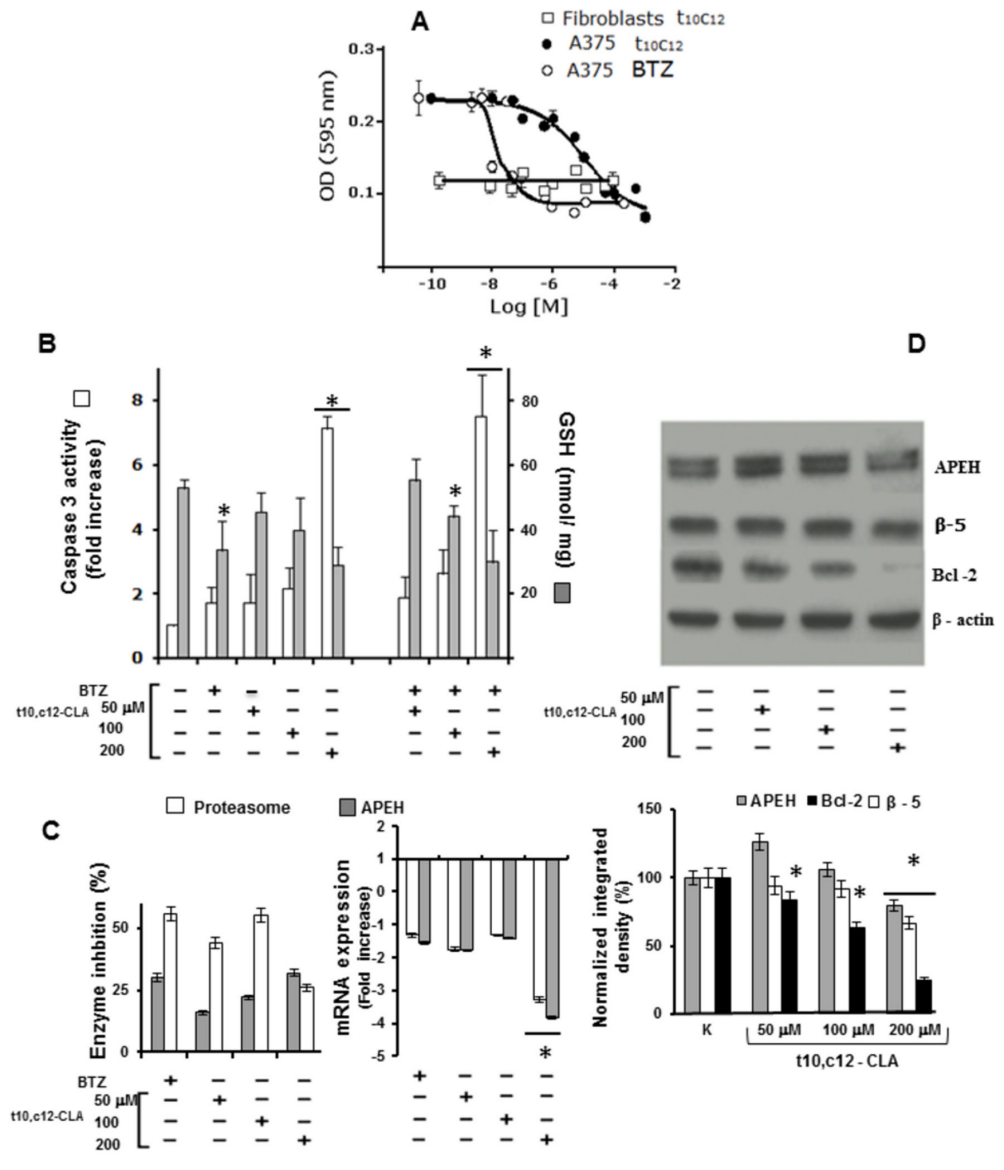
Dose-dependent pro-apoptotic activity of t10,c12-CLA correlates with down-regulation of GSH, APEH and proteasomal CT-like subunit at both mRNA and activity level in A375 cells. Isobologram of A375 cells treated with t10,c12-CLA or BTZ for 24h is reported in panel A. Human fibroblasts exposed to the same t10,c12-CLA concentrations were used as control. Data are expressed as means ±SD values of triplicate data from three independent experiments. Pre-confluent A375 cultures were incubated for 24h with 50, 100 or 200 µM t10,c12-CLA. Thereafter, cells were harvested, and used for cytoplasmic or mRNA extracts preparation. Cells untreated or treated with 10 nM BTZ were used as negative or positive controls, respectively. Measurement of GSH concentration (B), caspase 3 activity (B) and APEH or proteasomal CT-like activities (C left panel) were performed on cytoplasmic extracts. The mRNA levels of APEH and β-5 subunit were evaluated by qRT-PCR and expressed as fold change in comparison to untreated cells (C right panel). Intracellular levels of Bcl-2, APEH and β-5 were detected by immunoblotting (D upper panel). Data on Western blot analysis were normalized to the density of control (β-actin) and the values were expressed as percent value as compared to untreated cultures (K) on triplicate measurements (D lower panel). Results were presented as means ±SD of triplicate data from three independent experiments. *Significantly different (P < 0.01) from respective controls.

In order to define the dose accountable for 50% decrease of cell viability (IC_50_), A375 cells were exposed for 24h to a concentration range of t10,c12-CLA or BTZ (from 10 nM to 400 μM), using human fibroblasts as control. The resulting isobologram revealed that the IC_50_ values were 1.0±0.02 μM or 10.0±0.02 nM for t10,c12-CLA or BTZ, respectively. Moreover, proliferation data obtained from fibroblasts, even at higher concentration of t10,c12-CLA, further supported the lack of toxic effects (Figure 5A). 

Next, cultures were incubated with increasing t10,c12-CLA doses (50, 100 or 200 μM) and the possible additive effect elicited by sub-toxic amount of BTZ (5 nM) was evaluated in cells co-incubated with t10,c12-CLA for 24h. The results obtained (Figure 5B) demonstrated that the dose-dependent activation of caspase 3 was triggered by t10,c12-CLA, reaching an eightfold increase compared to the control culture. Notably, pro-apoptotic induction, associated with a significant decline in intracellular GSH, was not further improved by BTZ supplementation (Figure 5B). Similarly, APEH and proteasome mRNA levels were strongly down-regulated by 200 μM t10,c12-CLA treatment (Figure 5C, right panel) and only minor alterations were produced by the addition of BTZ (data not shown). Interestingly, while a dose-dependent inhibition of APEH activity was observed, the proteasomal CT-like activity was inhibited to 46 and 50% by 50 and 100 μM t10,c12-CLA, respectively and a less marked effect resulted from cells exposed to 200 μM CLA (25%) (Figure 5C, left panel). Moreover, the decline of APEH and β-5 protein expression only occurred at the higher CLA dose (p<0.05) (Figure 5D). In addition, the noticeable decrease of the anti-apoptotic protein Bcl-2 expression, reaching the maximum reduction of 80%, further supported the role of apoptosis in the anti-proliferative effect of t10,c12-CLA (Figure 5D). Finally, we showed that cell exposure to high t10,c12-CLA doses markedly down-regulated the Nrf2 pathway, as evidenced by the declined mRNA levels of some target genes (NQO1 and γGCL), expressed as fold change in comparison to untreated cells (Figure S2). 

These findings support the hypothesis that the combined down-regulation of antioxidant/detoxifying defences, APEH/proteasome system and Bcl-2 levels, may play an important role in apoptosis induction triggered by t10,c12-CLA in A375 cells. 

### A375 exposure to high t10,c12-CLA doses increases ROS production in association with apoptotic events and APEH/proteasome down-regulation in time-dependent fashion

**Figure 6:**
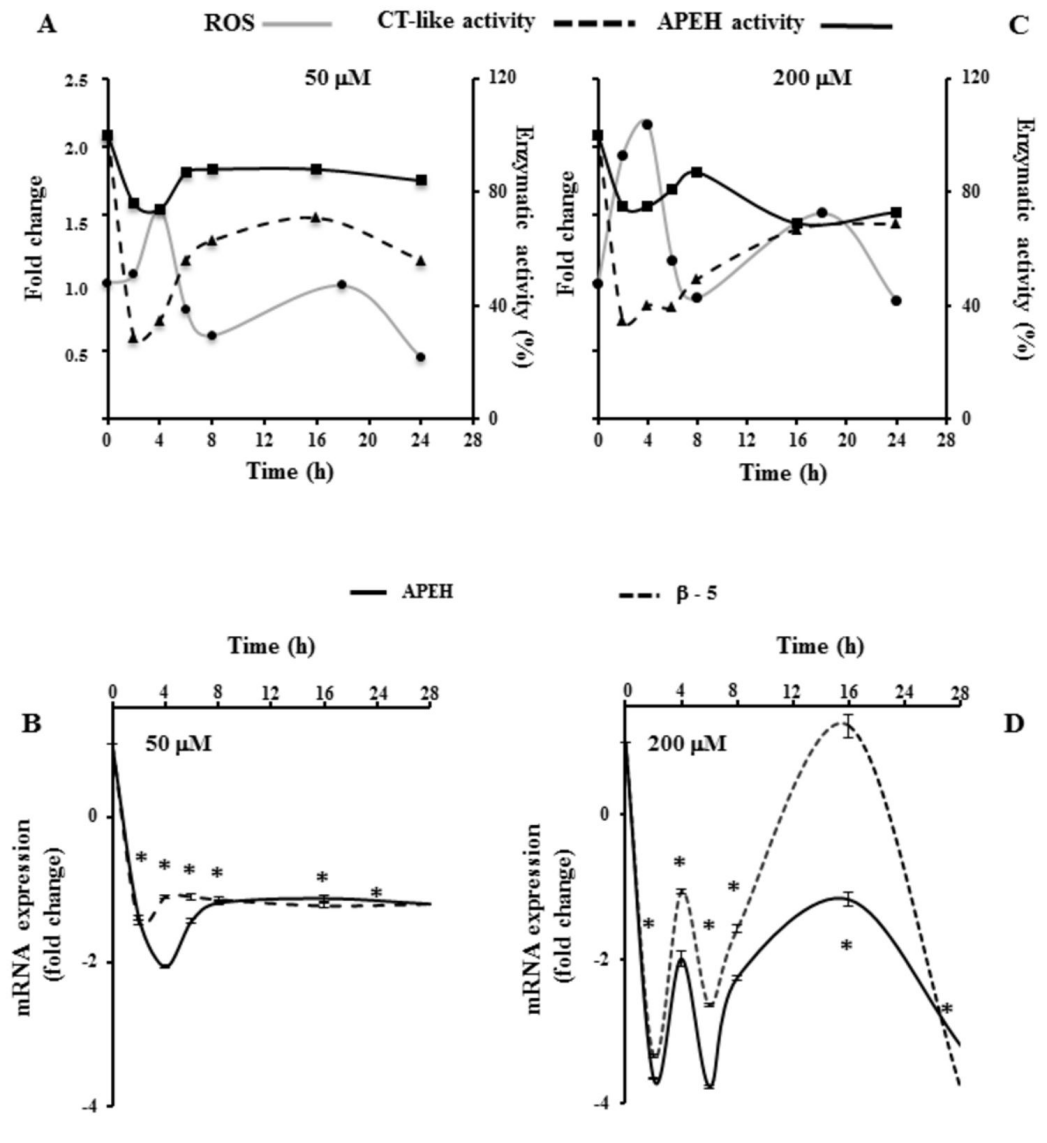
Time-dependent effects of t10,c12-CLA on APEH/proteasome system and on ROS production in A375 cells. Pre-confluent A375 cells were incubated with 50 μM or 200 μM of t10,c12-CLA for the indicated times. After treatments, cytoplasmic cell-extracts were used for the measurement of APEH and proteasomal CT-like activities (A,C). The ROS profiles were compared with the time courses of proteasomal CT-like and APEH activity levels (A,C). ROS production was assessed as described in materials and methods. cDNAs were synthesized and used for qRT-PCR amplification of APEH and β-5 (B,D) at the indicated times. The mRNA levels were finally expressed as fold change in comparison to untreated cells. Results were presented as means ±SD of triplicate data from three independent experiments and SD values lower than 5% were not shown. *Significantly different (P < 0.01) from respective controls.

 Time-dependent monitoring of ROS production, APEH and proteasome (β-5) at mRNA and enzyme activity level, was performed to evaluate the effects produced by the exposure to lower (50 μM) or higher (200 μM) t10,c12-CLA concentrations, on pre-confluent A375 cells. Sudden decrease (2h) of APEH and proteasomal CT-like activities in cells exposed to low doses, correlated with a transient reduction of their mRNA expression. Upon this early response, enzyme activities recovered, reaching a plateau after 8h with values corresponding to 80 or 70% of their starting values, respectively (Figure 6A). Similarly, mRNA profiles showed a short-lived gene repression, which quickly recovered towards the stable final values, being approximately one-fold lower than their initial expression level (Figure 6B). Conversely, the higher concentration of t10_,_c12-CLA produced a downshift of APEH activity reaching a plateau with average values of 70% compared to its starting level, whereas a long-term down-regulation of proteasomal activity persisted up to 16h (Figure 6C). Interestingly, two transient minima of mRNA levels were observed after 2 and 6h, followed by a significant increase until 16h. After 24h of incubation, APEH and β-5 expression decreased again reaching the corresponding lowest values (Figure 6D).

The time-dependent ROS production indicated that the early down-regulation of APEH /proteasome enzyme activities could be induced by ROS yield (Figure 6A), while a direct modulation of the CLA isomer on both enzymes can possibly contribute to the following decrease of the activity/mRNA levels observed at 200 μM (Figure 6C,D). Cell pre-incubation with the antioxidant N-acetyl cysteine (NAC, 5 mM) before the 200 μM CLA exposure (2 or 24h) resulted in a marked cytotoxic effect (data not shown). 

**Figure 7:**
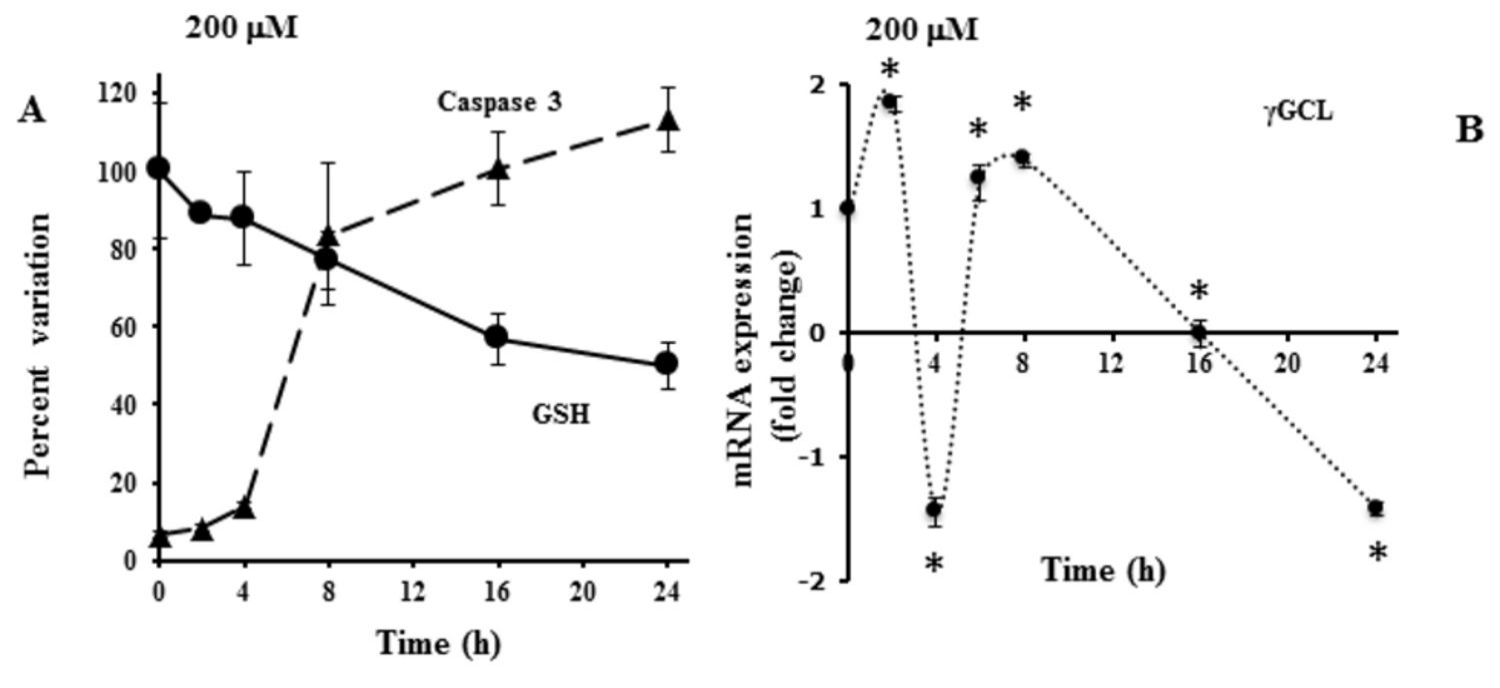
Time-dependent effects of t10,c12-CLA on caspase 3 and cyto-protective defences on A375 cell. Pre-confluent A375 cultures were incubated with 200 μM t10,c12-CLA for the indicated times. After treatments, cytoplasmic cell-extracts were used for the measurement of GSH concentration and caspase 3 activity (A). GSH and caspase 3 activities were expressed as percent variation in comparison to cells harvested at the beginning or at the end of the incubation, respectively. cDNAs were synthesized and used for qRT-PCR analysis of γGCL transcripts (B) at the indicated times. The mRNA levels were finally expressed as fold change in comparison to untreated cells. Results were presented as means ±SD of triplicate data from three independent experiments. SD values lower than 5% were not shown. *Significantly different (P < 0.01) from respective controls.

Finally, time-dependent effects elicited by 200 μM t10,c12-CLA on GSH concentration and caspase 3 activity, together with γGCL mRNA levels, were measured. As shown in Figure 7A, the decline of intracellular GSH was followed by caspase 3 activation (after 6-8h). To investigate the mechanism underlying the pro-oxidant activity of t10,c12-CLA, the mRNA expression of the rate-limiting enzyme responsible for cellular GSH synthesis (namely γGCL) was monitored. As expected, the early activation triggered by CLA isomer (after 2h) was followed by a transient decrease in mRNA (peaking after 4h), which temporarily recovered before leading to the down-regulation (1.5 fold) of mRNA levels (Figure 7B). 

## Discussion

Owing to their enhanced metabolic activity, cancer cells require elevated levels of energy to maintain a high rate of cell growth and proliferation. This is also guaranteed by an improved activity of the Ubiquitin-Proteasome System, which is the major pathway for protein turnover in eukaryotes [29], providing a secondary antioxidant defence mechanism, in combination with APEH [15,16]. Indeed, protein homeostasis is critically involved in cancer cell survival; thus, one of the major *focus* in cancer research is targeting the balance between the production and destruction of proteins mediating cell proliferation. In this context, proteasome inhibition represents a novel strategy against many tumoral diseases, triggering an increase in apoptosis and decrease in cellular growth. Accordingly, in the last decade, research and development of new compounds able to down-regulate proteasome functions have attracted growing attention. 

It is known that the pro-apoptotic ability of CLA mixture (c9,t11- and t10,c12-CLA; 50:50) or its individual isomers, affects tumor cell proliferation *via* different biochemical pathways involving apoptotic or survival genes (Bcl-2, p21, p53). The efficacy of these isomers in inhibiting the cancer cell viability was highly influenced by the model system used, within a concentration range of 1-200 μmol/L and treatment lasting 1-11 days [18]. Specifically, t10,c12-CLA has revealed a more efficient activity, respect to c9,t11-CLA isomer, in modulating apoptosis or cell cycle. In human prostatic carcinoma cells, t10,c12-CLA anticancer effect associates to decreased Bcl-2 and increased p21(WAF1/Cip1) mRNA levels [30] while in human colon or bladder cancer cells it was accompanied by the activation of ATF/NAG-1 [31] or Insulin Growth Factor signaling [32]. Moreover, it was reported that t10,c12-CLA was able to down-regulate Fatty Acid Synthase [33] or antioxidant defence systems [21,22,34] in different human cancer cells. 

In such a context, the purpose of this study was to explore the relationship between the anti-proliferative properties and the ability of CLA isomers to down-regulate the APEH/proteasome system in cancer cells, taking into account the role of cellular redox status in these processes. 

We firstly evaluated the effects of CLA isomers on purified proteasomes and APEH in *cell free* assays, showing that t10,c12-CLA was the only isomer able to efficiently inhibit both enzymes, which appeared functionally correlated, in a cancer cell panel. 

Intriguingly, the link observed between caspase 3 activation and cell viability in t10,c12-CLA treated cells, supported the apoptosis role in the anti-proliferative effects specifically induced by this isomer. The higher susceptibility to the t10,c12-CLA treatment of A375 melanoma cell line, showing the highest basal levels of APEH/proteasome, is consistent with the involvement of this system in cell survival. Unfortunately, this hypothesis cannot be extended to all the tested cell lines showing high constitutive enzymatic levels. In addition, we demonstrated that early ROS production triggered by higher t10,c12-CLA doses, along with the combined down-regulation of NF-E2-related factor 2-Antioxidant responsive elements (Nrf2-ARE) pathway and proteasome-APEH activity/expression levels, was likely responsible for the programmed A375 cell death. However, these results couldn’t be further investigated by using antioxidants (NAC) and t10,c12-CLA combination in cell treatment (data not shown) due to NAC toxicity on A375 cells [35]. 

The endogenous oxidative stress rarely leads to damage, because a healthy cell generally possesses a powerful antioxidant defence to inactivate ROS. However, when cellular antioxidants are compromised, as occurs in the context of external environmental challenges, cell death is the expected outcome. By contrast, in several tumoral cells, hyperactivation of endogenous sources of ROS, which generates the observed increased levels of these molecules, results in a state of chronic oxidative stress [2,15]. It is well established that GSH plays an important role in cancer development and treatment, as it can protect against DNA damages produced by ROS and electrophilic chemicals [36]. Generally, in various types of cancerous cells and solid tumors, elevated GSH levels are observed, making these cells and tissues less susceptible to chemotherapy by increasing the resistance to oxidative stress. However, although chronic ROS exposure confers several advantages to cancer cells, by stimulating proliferation and maintaining the transformed phenotype [37], excessive ROS yield may induce cell cycle arrest and apoptosis. Therefore, redox state modulation in tumoral cells has been indicated as a possible target for cancer [38] or, specifically, for melanoma treatment [39]. 

In this context, our results, showing the increased intracellular GSH levels in A375 cells, were in agreement with studies reporting the central role played by redox homeostasis in the control of melanoma survival, proliferation and invasiveness [40]. Moreover, the association of pro-oxidant activity of t10,c12-CLA with anti-proliferative effect, was consistent with literature [21,22] and conformed to the activities of recently discovered proteasome inhibitors, triggering ROS production in melanoma cells through oxidative stress activation [41,42]. In addition, although the down-regulation of the Nrf2 pathway, was accompanied by the caspase 3 activation in cells exposed to high t10,c12-CLA doses , nevertheless there is not necessarily a direct cause/effect between these two events. In accordance with the importance of Nrf2 down-regulation in tumour growth reduction and in enhancing the efficacy of chemotherapeutic agents [43], the use of t10,c12-CLA in combination with specific APEH/proteasome inhibitors could represent an effective strategy for melanoma treatment. 

**Figure 8:**
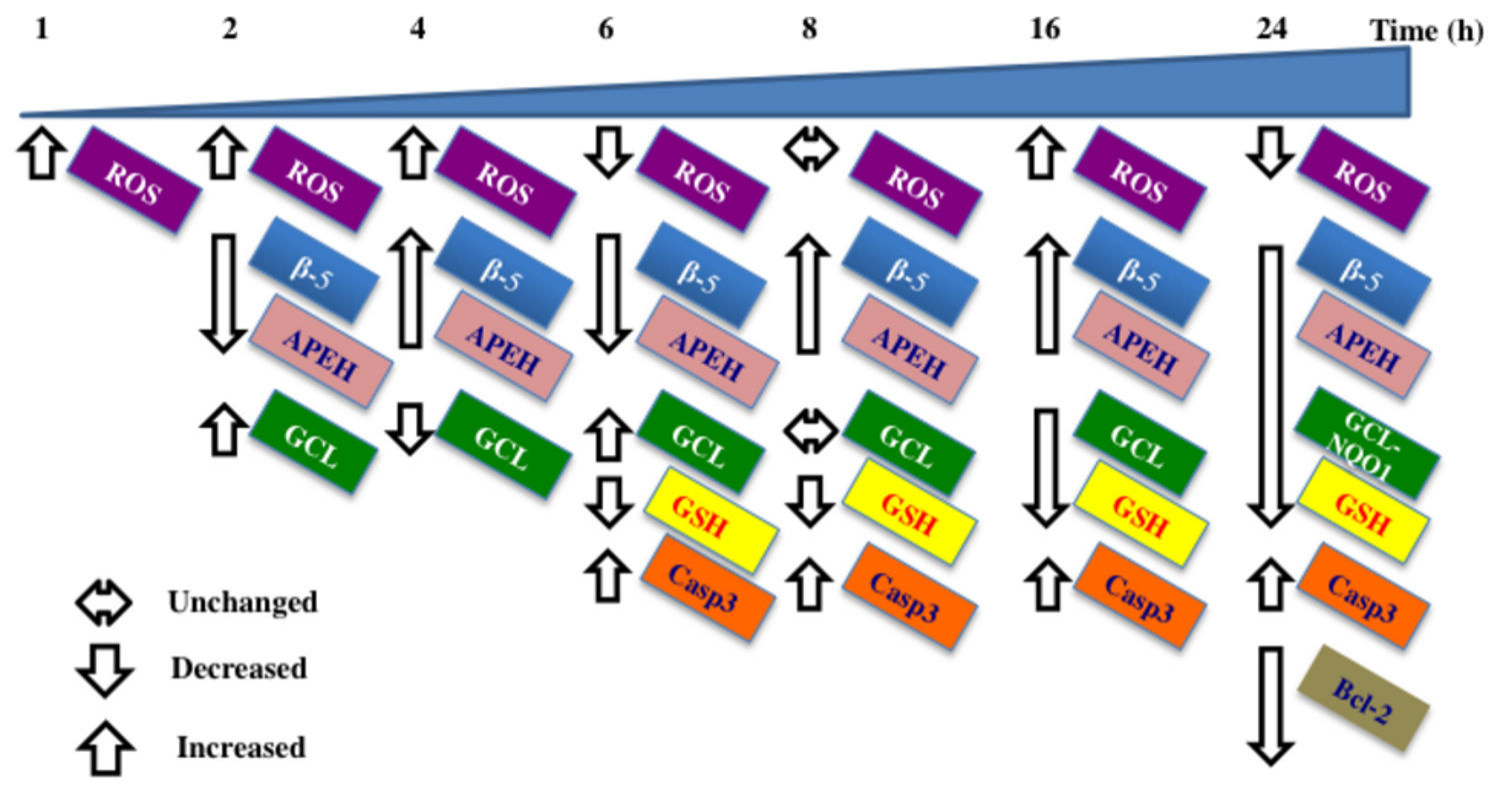
Summary diagram. In the scheme early ROS yield (after 1h), triggered by cells exposure to 200 μM t10,c12-CLA, led to the transient decline of the detoxifying APEH/proteasome system and the improved γGCL expression, following the increased nuclear translocation of Nrf2. During the next 10h, the partial recovery of β-5 and APEH transcription paralleled the reduced GCL expression and intracellular GSH levels resulting in the increased apoptosis (caspase 3 activity, casp3). After 24h incubation, the simultaneous decline of β-5, APEH, γGCL and NQO1 transcriptional levels and of intracellular GSH are associated with decreased cell viability likely *via* apoptosis enhancement (as evidenced by increased casp3 activity and Bcl-2 degradation).

To sum up, t10,c12-CLA-induced oxidative stress was detectable at very early times, as revealed by the increase of DCF fluorescence (Figure **6C**), down-regulation of γGCL expression (Figure **7B**) and the following decline of intracellular thiols (Figure **7A**). Hence, it is reasonable to hypothesize that the oxidative stress and the Nrf2-activation, triggered by t10,c12-CLA, are upstream processes contributing to the APEH/proteasome down-regulation (Figure **8**) [11,44,45] which culminate in activation of caspase 3. 

The finding of time progression events provides additional insights toward understanding the CLA-activated mechanisms, which are involved in the anticarcinogenic effects of these compounds, particularly the t10,c12-CLA isomer, in melanoma cancer cells. Further research are needed to support the role played by APEH in the down-regulation of cancer cell viability.

## Supporting Information

Figure S1 **Proteasome activity is differently down-regulated by CLA isomers.** Proteasomal CT-like activity was measured in eight cancer cell lines exposed to 200 µM of t10,c12- (dark grey bars) or c9,t11-CLA (light grey bars). Cell cultures exposed to octanoic acid (200 µM, black bars) or to BTZ (10 nM, white bars) were used as negative or positive controls, respectively. Data are expressed as means ±SD values of triplicate data from three independent experiments, SD values lower than 5% were not shown.
(PDF)

Figure S2 **mRNA levels of GCL and NQO1 in A375 cells treated with 50 or 200 μM of t10,c12-CLA for 24h.** The mRNA levels were evaluated by RT-PCR and expressed as fold change in comparison to untreated cells. *Significantly different (P < 0.01) from respective controls.
(PDF)
