# Supplementary material for: RedOx Status, Proteasome and APEH: Insights into Anticancer Mechanisms of t10,c12-Conjugated Linoleic Acid Isomer on A375 Melanoma Cells
Source: PLoS One. 2013 Nov 19;8(11):e80900. doi: 10.1371/journal.pone.0080900 (PMC3834215; doi:10.1371/journal.pone.0080900)
Supplement: Figure S1 — Proteasome activity is differently down-regulated by CLA isomers. Proteasomal CT-like activity was measured in eight cancer cell lines exposed to 200 µM of t10,c12- (dark grey bars) or c9,t11-CLA (light grey bars). Cell cultures exposed to octanoic acid (200 µM, black bars) or to BTZ (10 nM, white bars) were used as negative or positive controls, respectively. Data are expressed as means ±SD values of triplicate data from three independent experiments, SD values lower than 5% were not shown. (PDF) [file pone.0080900.s001.pdf]

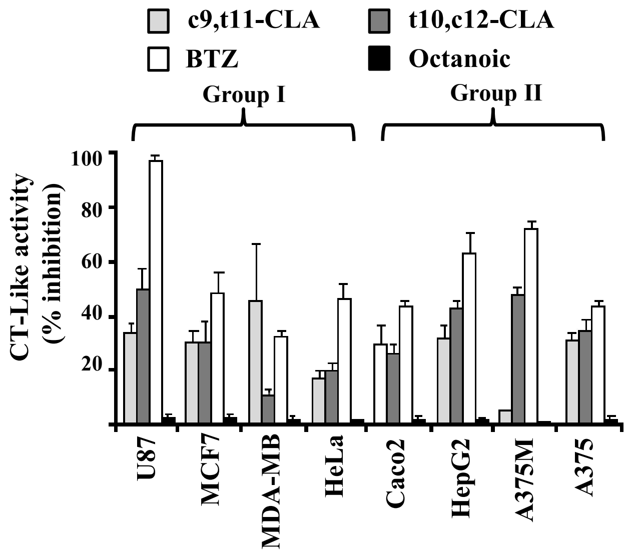

Supplementary Figure S1. Proteasome activity is differently down-regulated by CLA isomers. Proteasomal CT-like activity was measured in eight cancer cell lines exposed to 200 µM of t10,c12- (dark grey bars) or c9,t11-CLA (light grey bars). Cell cultures exposed to octanoic acid (200 µM, black bars) or to BTZ (10 nM, white bars) were used as negative or positive controls, respectively. Data are expressed as means ±SD values of triplicate data from three independent experiments, SD values lower than 5% were not shown.
