# Supplementary material for: RedOx Status, Proteasome and APEH: Insights into Anticancer Mechanisms of t10,c12-Conjugated Linoleic Acid Isomer on A375 Melanoma Cells
Source: PLoS One. 2013 Nov 19;8(11):e80900. doi: 10.1371/journal.pone.0080900 (PMC3834215; doi:10.1371/journal.pone.0080900)
Supplement: Figure S2 — mRNA levels of GCL and NQO1 in A375 cells treated with 50 or 200 μM of t10,c12-CLA for 24h. The mRNA levels were evaluated by RT-PCR and expressed as fold change in comparison to untreated cells. *Significantly different (P < 0.01) from respective controls. (PDF) [file pone.0080900.s002.pdf]

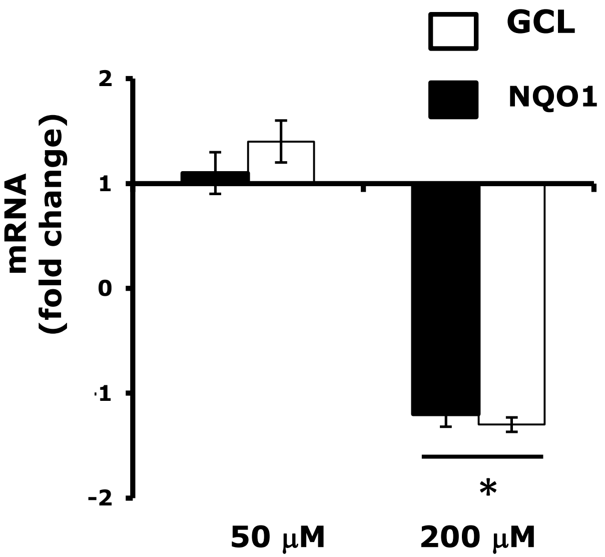

Supplementary Figure S2. mRNA levels of GCL and NQO1 in A375 cells treated with 50 or 200 μM of t10,c12-CLA for 24h. The mRNA levels were evaluated by RT-PCR and expressed as fold change in comparison to untreated cells. *Significantly different ($P < 0.01$) from respective controls
